# Potential and limitation of air pollution mitigation by vegetation and uncertainties of deposition-based evaluations

**DOI:** 10.1098/rsta.2019.0320

**Published:** 2020-09-28

**Authors:** Eiko Nemitz, Massimo Vieno, Edward Carnell, Alice Fitch, Claudia Steadman, Philip Cryle, Mike Holland, R. Daniel Morton, Jane Hall, Gina Mills, Felicity Hayes, Ian Dickie, David Carruthers, David Fowler, Stefan Reis, Laurence Jones

**Affiliations:** 1 UK Centre for Ecology and Hydrology, Penicuik EH26 0QB, UK; 2 UK Centre for Ecology and Hydrology, Environment Centre Wales, Bangor LL57 2UW, UK; 3 School of GeoSciences, University of Edinburgh, Edinburgh EH8 9XP, UK; 4 EFTEC, Economics for the Environment Consultancy, London, UK; 5 ERMC, Reading, UK; 6 UK Centre for Ecology and Hydrology, Lancaster Environment Centre, Lancaster, UK; 7 Cambridge Environmental Research Consultants Ltd (CERC), Cambridge, UK; 8 European Centre for Environment and Health, University of Exeter Medical School, Truro, UK; 9 Department of Geography and Environmental Science, Liverpool Hope University, Liverpool, UK

**Keywords:** i-Tree Eco, green infrastructure, nature-based solutions, dry deposition

## Abstract

The potential to capture additional air pollutants by introducing more vegetation or changing existing short vegetation to woodland on first sight provides an attractive route for lowering urban pollution. Here, an atmospheric chemistry and transport model was run with a range of landcover scenarios to quantify pollutant removal by the existing total UK vegetation as well as the UK urban vegetation and to quantify the effect of large-scale urban tree planting on urban air pollution. UK vegetation as a whole reduces area (population)-weighted concentrations significantly, by 10% (9%) for PM_2.5_, 30% (22%) for SO_2_, 24% (19%) for NH_3_ and 15% (13%) for O_3_, compared with a desert scenario. By contrast, urban vegetation reduces average urban PM_2.5_ by only approximately 1%. Even large-scale conversion of half of existing open urban greenspace to forest would lower urban PM_2.5_ by only another 1%, suggesting that the effect on air quality needs to be considered in the context of the wider benefits of urban tree planting, e.g. on physical and mental health. The net benefits of UK vegetation for NO_2_ are small, and urban tree planting is even forecast to increase urban NO_2_ and NO*x* concentrations, due to the chemical interaction with changes in BVOC emissions and O_3_, but the details depend on tree species selection. By extrapolation, green infrastructure projects focusing on non-greenspace (roadside trees, green walls, roof-top gardens) would have to be implemented at very large scales to match this effect. Downscaling of the results to micro-interventions solely aimed at pollutant removal suggests that their impact is too limited for their cost–benefit analysis to compare favourably with emission abatement measures. Urban vegetation planting is less effective for lowering pollution than measures to reduce emissions at source. The results highlight interactions that cannot be captured if benefits are quantified via deposition models using prescribed concentrations, and emission damage costs.

This article is part of a discussion meeting issue ‘Air quality, past present and future’.

## 1. Introduction

### (a) Motivation

Air pollution is considered a key environmental threat to human health. The World Health Organisation (WHO) attributes some 7 million premature deaths to ambient and indoor air pollution annually, with many of these occurring in the urban centres of the developing world [1,2]. Few countries achieve WHO air quality guideline values, recommending, e.g. PM_2.5_ concentrations <10 µg m^−3^ and NO_2_ <40 µg m^−3^ at the annual mean, and instead, interim air quality policy targets have been adopted that provide a realistic medium-term ambition and promote progress towards cleaner air. For example, as emissions have decreased across Europe, successive editions of the European Air Quality Directive [3] and national implementations have set increasingly stringent Air Quality (AQ) objectives that have continued to drive improvements in air quality and motivated the implementation of increasingly stricter emission standards to achieve these objectives. However, despite air quality improving in many developed countries in the past decades [4], both developing and developed countries still struggle to adhere to the AQ objectives whether set by themselves or in international negotiations, based on the WHO recommendations.

Over the past years, there have been setbacks in the attempts to reduce emissions. A prominent example is the failure of many diesel vehicles complying with EURO5 and early EURO6 European emission standards to achieve the required reduction in NO*x* emissions under real-world driving conditions [5]. In other cases, especially in the developing world, gains of increased emission control have been offset by increased activity, e.g. car ownership and annual distance travelled (e.g. [6]). As legal AQ targets are being missed through emission control, national, regional and local governments are forced to look for alternative interventions for reducing air concentrations and human exposure. This includes the introduction of low emission zones and increasingly also the targeted use of vegetation to lower concentration levels. In the UK, several local authorities have embarked on efforts to increase urban forest cover in recent years, with air pollution reduction being one of the motivators. At the same time, there is an increasing interest in valuing the air pollution removal by vegetation [7,8] as part of the overall ecosystem service it provides and to include this valuation in national accounts.

In this paper, we investigate the effectiveness of existing vegetation and potential green infrastructure interventions in relation to their spatial scale and critically assess the cost/benefit of micro-interventions by considering the basic physical constraints on pollutant uptake. The paper quantifies the effect of UK-wide vegetation on lowering air pollution levels and assesses the current role of UK urban vegetation as a whole. This work was done as part of the UK national accounts compiled by the UK Office for National Statistics [9]. The paper continues, by looking at the potential effects of large-scale city-wide interventions, converting significant fractions (25 and 50%) of the available urban open greenspace into urban woodland, again for the UK. It then scales down the results to discuss the potential of smaller green infrastructure interventions and the effect they could possibly have on air pollution levels, with focus on the urban environment.

### (b) Approaches for quantifying pollutant removal by vegetation at large spatial scales

#### (i) The deposition-based approach to quantifying forest ecosystem services

Several approaches have been used in the past to quantify pollutant removal by extensive vegetation areas and to value this ecosystem service. Most of these value the benefit of pollutant removal via the equivalent emission damage costs, i.e. the same damage cost that governments ascribe to emissions of pollutants can be used to valued air pollution removal (as a negative emission). These emission damage costs have been tabulated from offline calculations using atmospheric chemistry and transport models (ACTMs) to assess the consequences of the emissions in terms of population exposure and outcomes [10]. They often vary by degree of urbanization reflecting the proximity and size of exposed population.

The pollutant removal is then quantified with models such as the Urban Forest Effects Model (UFORE) [11] and its descendant i-Tree Eco [12], which is based on modelling the uptake of individual trees. While computationally simple, there are several disadvantages to this method: (i) the approach is indirect in its evaluation as it relies on modelling of emission–exposure relationships to quantify the underlying emission damage costs in the first place, (ii) it is not mass conserved in the sense that it is driven with prescribed concentrations with no feedback of the deposition on concentrations, and (iii) it, therefore, does not take other interactions and processes into account that respond to concentration changes such as atmospheric chemistry and wet deposition. By contrast, one advantage is that the deposition-based approach allows for a detailed description of the vegetation, in the case of i-Tree Eco the trees or tree canopy.

#### (ii) Atmospheric chemistry and transport modelling

Full application of a three-dimensional ACTM overcomes the shortcomings described above, but in turn tends to rely on a simplified representation of the vegetation during the analysis and its spatial resolution is limited to the model grid size, in this case approximately 5 × 6 km^2^. An ACTM tracks the pollutant emissions, their chemical transformations and deposition in a three-dimensional meteorological (flow) field. Rather than prescribing the pollutant concentrations, they are predicted by the model as influenced by the dry deposition to the underlying landcover in the model. With this modelling framework, the effect of vegetation can be quantified by comparing two model runs with modified landcover description. This approach not only quantifies the additional dry deposition induced by the vegetation, but also directly the influence on the concentration. From this concentration field, human exposure and health impacts can be derived more directly than via the emission damage costs. In addition, this approach accounts for the full range of chemical and spatial interaction. For example, the enhanced removal of PM precursor gases (NH_3_, SO_2_, NO_2_, VOCs) and also oxidants (e.g. O_3_) by vegetation reduces their contribution to secondary PM formation and thus reduces PM_2.5_ through a route that is additional to enhanced PM_2.5_ dry deposition.

A crucial additional step for this approach is the decision on the baseline scenario: removal-based approaches such as iTrees Eco normally derive the benefit from the quantification of the dry deposition to the vegetation, without consideration that some, although reduced, dry deposition would also occur to a non-vegetated surface. In the ACTM approach, the non-vegetated landcover needs to be prescribed explicitly and the choice affects the results.

This paper uses this ACTM-based approach to evaluate the effect of vegetation and in doing so also quantifies explicitly some of the interactions in the model that would be missed with the deposition-based approach.

## 2. Methods

### (a) Modelling the impact of vegetation on air pollution at large spatial scales

In this study, an implementation of the European EMEP Eulerian ACTM [13] was used with a nested higher resolution UK domain at a scale of approximately 5 × 6 km^2^ (EMEP4UK) [14–17]. The current baseline reference (UKBASE) UK landcover definition was derived by remapping the UKCEH Landcover Map 2007 (LCM2007) [18] to the seven existing landcover classes of the EMEP model (deciduous forest, coniferous forest, crops, semi-natural land, water, desert and urban). For the urban scenario runs, LCM2015 was used and three new landcover classes (urban forest, urban open greenspace, urban water) were derived from the Ordnance Survey MasterMap for areas lying within the urban morphology layer of the UK as described in detail by Jones *et al*. [8]. Briefly, first land was classified as ‘urban’ based on the existing urban morphology layer from the UK Office for National Statistics, supplemented by a variable buffer. In a second step, this urban layer was intersected with the OSMasterMap ‘natural surface’ category, and of the resulting urban vegetation map all OSMasterMap objects with the term ‘trees’ or ‘woodland’ in the main descriptor were assigned to the ‘urban forest’ class and the remainder to the ‘urban open greenspace’ class, which thus contains e.g. open parkland, gardens and playing fields.

The EMEP4UK model uses a tiled approach, in which the landcover fractions of the seven types are specified for each grid cell. The meteorological input was generated with the community weather research and forecasting (WRF) model v. 3.7.1 (www.wrf-model.org) which included data assimilation (Newtonian nudging) of the numerical weather prediction (NWP) model to the meteorological reanalysis from the US National Center for Environmental Protection (NCEP)/National Center for Atmospheric Research (NCAR) Global Forecast System (GFS) at 1° resolution [19] at 6-hourly intervals. The performance of this model combination (WRF-EMEP4UK) for the UK has been thoroughly established elsewhere [14–17].

Alternative landcover maps were created: in the first, no-vegetation (NoVEG) scenario, all vegetation landcover (i.e. deciduous, coniferous, crops and semi-natural; urban and non-urban) within the UK was replaced by desert, representing bare soil. In addition, a no-urban-vegetation (NoUrbanVEG) landcover map was created by replacing only urban vegetation (urban forest and open urban greenspace) with desert, and two tree planting scenarios were created by converting 25 and 50% of the open urban greenspace category into urban forest, referred to as 25OGSC and 50OGSC (open green space conversion), respectively. The total land area of the urban landcover classes in the various scenarios is summarized in table 1. Together, the urban landcover types represent 7.1% of the UK land area.

**Table 1.** Summary of the landcover statistics of the various urban planting scenarios (water bodies not included), stating the land area for each landcover class with their fractional contribution to total urban landcover in parentheses.

	status quo ‘UrbanBASE’ (km^2^)	no urban vegetation ‘NoUrbanVEG’ (km^2^)	25% planting ‘25OGSC’ (km^2^)	50% planting ‘50OGSC’ (km^2^)
urban woodland	976 (5.5%)	0	2007 (11.4%)	3038 (17.2%)
open urban greenspace	4124 (23.4%)	0	3093 (17.5%)	2062 (11.7%)
urban bare soil	0 (0%)	510.0 (30.0%)	0 (0%)	0 (0%)
urban sealed	12 362 (70.0%)	12 362 (70.0%)	12 362 (70.0%)	12 362 (70.0%)

The effect of the choice of the baseline scenario/non-vegetated landcover has implications for the results. Theoretical choices could include sealed land (concrete/asphalt) or bare soil. In the EMEP modelling system, only two terrestrial non-vegetated terrestrial landcover types exist: urban (with the aerodynamic roughness of the built-up environment) and desert (with the properties of sand). Under the assumption that desert comes closest to bare soil in terms of aerodynamic properties and its affinity for pollutant uptake, but that its grain size distribution results in large resuspension, a decision was made to use desert as a reference, but to completely discount the contribution of desert dust to PM_2.5_ in all runs and/or to report it separately. It should be borne in mind, however, that some of the effect of increased resuspension is real.

The model set-up and landcover scenario use is summarized in table 2. The UK vegetation simulations were carried out with EMEP4UK implementation of EMEP rv4.10, while the urban vegetation simulations were based on the more recent version EMEP rv4.17. Differences between the two versions are small, but one additional significant difference needs to be considered: in the UK vegetation simulations, soil NO emissions were calculated as a function of landcover and changed with vegetation cover, while in the urban vegetation simulations, NO emissions were prescribed and fixed between scenarios. Two different current-vegetation reference runs (UKBASE and UrbanBASE) were used to match the model set-ups of each set of simulations. All runs were performed with the same driving meteorology for the year in question, based on status quo landcover, i.e. WRF was not rerun for the various landcover scenarios, the rationale being that (i) the impact of landcover on the meteorology which drives advection (bottom layer is at 45 m) is a second-order effect and (ii) the modelled meteorology would then become incompatible with the observation-derived dataset against which it is constrained. In the tiled approach deployed for deposition in the EMEP model, the windspeed at a reference height is then extrapolated individually to the different landcover types, depending on landcover-specific roughness height and heat flux, weighted by the landcover scenarios.

The effect of current UK total vegetation cover on annual total pollutant deposition, annual average concentration fields and annual average human exposure was then derived by comparing an annual model run using the UKBASE or UrbanBASE landcover to a run based on the modified landcover scenarios. Effects were assessed for total PM_2.5_, SO_2_, NO_2_, O_3_ and NH_3_. UK national runs were performed for a range of meteorological years (2007, 2011 and 2015). For these baseline, gridded emissions of the UK National Atmospheric Emissions Inventory (NAEI) for 2014 were used for the 2015 run, and emissions for 2007 and 2011 were created by rescaling the 2014 gridded emissions according to the NAEI totals. All these UK runs were based on 2007 landcover. The urban runs were based on 2015 meteorology and the NAEI gridded emissions for 2015. Here, landcover was based on 2015.

**Table 2.** Summary of the scenario runs performed for this study.

scenario	WRF model version	EMEP model version	landcover scenario	soil NO emission
UK current vegetation (UKBASE)	3.7.1	4.10	UKCEH	landcover-dependent
			LCM2007	
UK no vegetation (NoVEG)	3.7.1	4.10	NoVEG	landcover-dependent
urban current vegetation (UrbanBASE)	3.7.1	4.17	UKCEH	prescribed
			LCM2015	
no urban vegetation (NoUrbanVEG)	3.7.1	4.17	NoUrbanVEG	prescribed
urban 25% open greenspace conversion (25OGSC)	3.7.1	4.17	25OGSC	prescribed
urban 50% open greenspace conversion (50OGSC)	3.7.1	4.17	50OGSC	prescribed

## 3. Results

### (a) Quantification of pollutant removal at the UK national scale

The total changes in PM concentrations are summarized for 2015 in table 3, with other years (2007 and 2011) shown in electronic supplementary material, table S1. Concentration and changes in concentrations were averaged both over the entire UK and over the predominantly urban grid cells (see the next section for details). In these summaries, the effect on the PM contribution from wind-blown dust has been listed individually. As mentioned above, due to the choice of desert as the reference surface, vegetation not only has the effect of capturing PM and its precursors, but also suppresses desert sand resuspension. The two effects are similar in magnitude for PM_2.5_ and the dust suppression is the larger effect for PM_10_, bearing in mind that resuspension shows large inter-annual variability, presumably linked to the statistics of extreme wind speeds and rainfall. Although vegetation does suppress resuspension from bare soil, desert sand is likely a poor proxy for UK soils when it comes to resuspension and the effect is likely overestimated by the model. For the non-dust PM components, vegetation lowers concentrations by about 10% for PM_2.5_ and 6% for PM_10_ as a UK spatial average, with the values being slightly smaller if averaged by population density. The benefit is predicted to have decreased with time both in absolute and relative terms (electronic supplementary material, table S1). Possible reasons are changes in the contribution of UK versus non-UK or primary versus secondary sources as emissions evolve over time. Biogenic secondary organic aerosol (BSOA) is the only component of the PM that is increased through the presence of vegetation. As a UK spatial average, about 55% of the BSOA is due to BVOC emissions from UK vegetation, the remainder presumably originating from BVOC sources outside the UK as the ACTM does not ascribe any BVOC emission to desert soil. As explained in §2a, values of PM_2.5_ and PM_10_ provided throughout the remainder of this paper do not include the wind-blown dust component.

**Table 3.** Annual average concentrations of PM under current and no-vegetation landcover scenarios and effect of change in concentration relative to national no vegetation scenario for 2015 meteorology (and 2014 emissions). Concentrations are in µg m^−3^ and averages are shown as UK area average, UK population-weighted (PW) average and as an area average over urban areas only. The urban area average is also derived for the national no-vegetation runs, but calculated over all grid cells for which the urban landcover types jointly account for at least 50%. wb dust: windblown dust from desert soil. BSOA: biogenic secondary organic aerosol.

pollutant	scenario	UK average	UK PW average	urban average
non-dust PM_10_	current vegetation (UKBASE)	9.90	11.77	11.84
	no vegetation (NoVEG)	10.55	12.53	12.60
	change in concentration	−0.65	−0.76	−0.77
	difference (%)	−6.15%	−6.09%	−6.07%
non-dust PM_2.5_	current vegetation (UKBASE)	4.85	6.69	6.79
	no vegetation (NoVEG)	5.40	7.35	7.46
	change in concentration	−0.55	−0.66	−0.67
	difference (%)	−10.2%	−9.03%	−8.94%
wb dust PM_10_	current vegetation (UKBASE)	0.11	0.097	0.10
	no vegetation (NoVEG)	2.00	1.73	1.87
	change in concentration	−1.89	−1.64	−1.77
	difference (%)	−94.6%	−94.4%	−94.4%
wb dust PM_2.5_	current vegetation (UKBASE)	0.026	0.023	0.025
	no vegetation (NoVEG)	0.46	0.40	0.43
	change in concentration	−0.44	−0.38	−0.40
	difference (%)	−94.3%	−94.1%	−94.2%
PM_2.5_ BSOA	current vegetation (UKBASE)	0.16	0.18	0.18
	no vegetation (NoVEG)	0.10	0.10	0.12
	change in concentration	+0.056	+0.076	+0.056
	difference (%)	+54.6%	+73.2%	+44.8%

**Figure 1.**
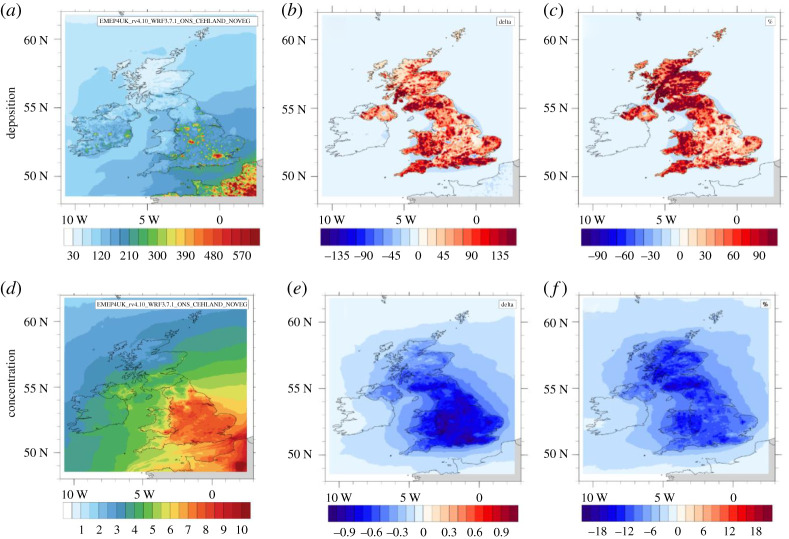
Model simulations for PM_2.5_ for 2015, showing (*a*) the annual total PM_2.5_ dry deposition to a vegetation-less UK (NoVEG) (mg m^−2^), together with the (*b*) absolute (mg m^−2^) and (*c*) relative (%) changes in deposition caused by the vegetation (UKBASE-NoVEG), with positive (red) values indicating an increase in deposition to vegetation compared with no vegetation. (*d*) The annual average PM_2.5_ surface concentration for a vegetation-less UK (µg m^−3^), together with its (*e*) absolute and (*f*) relative change due to vegetation (UKBASE-NoVEG), with negative (blue) values indicating a decrease in concentration above vegetation compared with no vegetation. The dust component is not included in these figures (see text).

The estimates of the change in dry deposition and surface concentration caused by the UK's total vegetation are shown in figure 1 for 2015 for PM_2.5_ (i.e. now without wind-blown dust) as an example. Urban areas show up in the deposition field of the no-vegetation run (figure 1*a*) not only because here PM_2.5_ concentrations are locally elevated, but also because urban buildings are aerodynamically rough and capture PM_2.5_ more effectively than smooth desert soils. The relative change in dry deposition between the two scenarios is particularly pronounced in the areas with woodland vegetation and elevated wind speeds because the dry deposition velocity (*V*_d_) of PM scales with wind speed in addition to being enhanced to forest.

**Figure 2.**
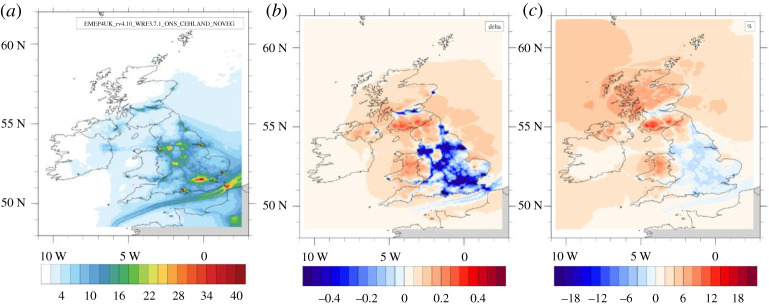
Model simulations for NO_2_ for 2015, showing (*a*) the annual average NO_2_ surface concentration for a vegetation-less UK (µg m^−3^), together with its (*b*) absolute (µg m^−3^) and (*c*) relative (%) change due to UK vegetation (UKBASE-NoVEG), with red (blue) values indicating an increase (decrease) in concentration above vegetation compared with no vegetation.

The effect of vegetation on the concentrations of the gaseous pollutants is summarized in table 4 for 2015 with additional years in electronic supplementary material, table S2. Spatially averaged relative reductions are largest for SO_2_ (30% overall), followed by NH_3_ (25%) and O_3_ (13%), with somewhat smaller reductions if weighted by population and for the urban grid cells. Surprisingly, the UK average effect is very small for NO_2_; the spatial pattern of figure 2 reveals that vegetation suppresses NO_2_ in source areas which is approximately balanced by increases in NO_2_ in rural areas, especially in areas with large forest cover. This is partly due to the fact that forest soils are associated with larger NO emissions than desert sand, but other interactions also contribute as also found in the urban landcover scenarios described below, in which NO emissions do not respond to landcover change and the reason is explored in §3c(iii).

**Table 4.** Average annual concentrations of a range of gaseous pollutants under current and no-vegetation landcover scenarios for 2015, and effect of change in concentration relative to no-vegetation scenario. Absolute concentrations are in µg m^−3^, and averages are shown as UK area average, UK population-weighted (PW) average and as an area average over urban areas only.

pollutant	scenario	UK area average	UK PW average	urban area average
SO_2_	current vegetation (UKBASE)	0.85	1.79	2.00
	no vegetation (NoVEG)	1.21	2.28	2.49
	change in concentration	−0.36	−0.49	−0.49
	difference (%)	−29.8%	−21.6%	−19.8%
NH_3_	current vegetation (UKBASE)	1.33	2.06	2.02
	no vegetation (NoVEG)	1.74	2.55	2.48
	change in concentration	−0.41	−0.49	−0.46
	difference (%)	−23.6%	−19.2%	−18.5%
NO_2_	current vegetation (UKBASE)	5.80	15.7	17.06
	no vegetation (NoVEG)	5.80	16.0	17.41
	change in concentration	0.00	−0.30	−0.35
	difference (%)	0.00%	−1.89%	−2.00%
O_3_	current vegetation (UKBASE)	70.58	64.68	64.04
	no vegetation (NoVEG)	82.83	74.69	73.33
	change in concentration	−12.24	−10.01	−9.29
	difference (%)	−14.8%	−13.4%	−12.7%

### (b) Quantification of total pollutant removal by UK urban vegetation and urban tree planting

**Figure 3.**
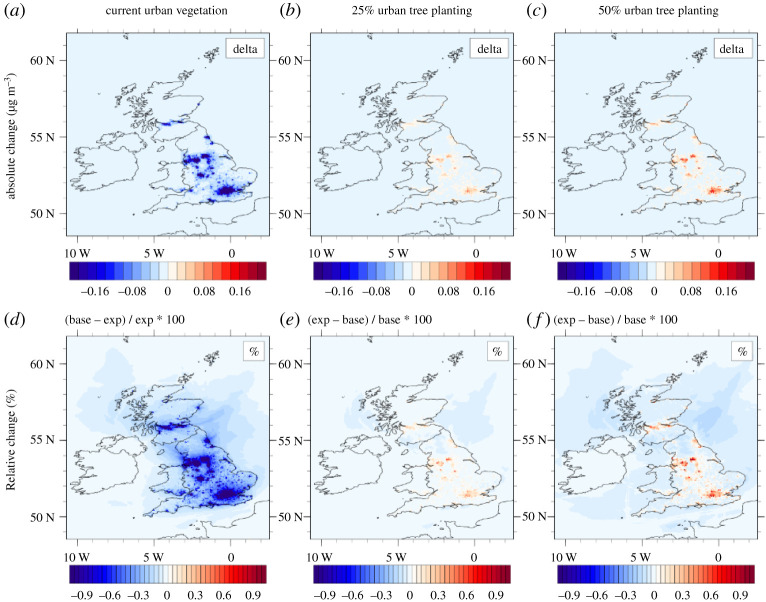
Maps for NO_2_ for the urban vegetation runs for 2015, showing the absolute (*a*) and relative (*d*) change in NO_2_ caused by current urban vegetation (UrbanBASE–NoUrbanVEG), as well as the associated results for the 25% (*b*,*e*) and 50% (*c*,*f*) urban woodland conversions (e.g. 25OGSC–UrbanBASE). The blue values in the current urban vegetation run (*a*,*d*) show the decrease in the concentrations caused by the present vegetation (relative to no vegetation). The red (blue) values in the tree planting scenario runs (*b*, *c*, *e* and *f*) indicate an increase (decrease) in the concentration due to the additional urban vegetation (relative to current urban vegetation).

Table 5 summarizes the effect of all current (2015) urban vegetation on annual average UK concentrations and the additional effect large-scale conversion from open urban greenspace to urban woodland could have. Also shown are the reductions averaged over all grid cells that are dominated by urban landcover, where most of the effect is expected and at which such intervention would be targeted. These ‘urban’ concentrations were very similar to UK population-weighted and urban population-weighted concentration changes which were explored as a further metric (electronic supplementary material, table S3). For PM_2.5_, the current urban vegetation is calculated to be responsible for reductions in PM_2.5_ of −0.95%, compared with −0.84 and −0.93%, depending on the averaging method. Overall, the model results indicate that current urban vegetation (5100 km^2^) reduces average urban PM_2.5_ concentration by about 1%, with a similar additional reduction (0.8%) expected from large-scale conversion of open urban greenspaces to additional woodland. The effect of existing vegetation is somewhat larger for the PM precursors SO_2_ and NH_3_, but the conversion to woodland has a comparably smaller effect than on PM because the deposition rate of the gases is less sensitive to the vegetation type. Interestingly, for both O_3_ and NO_2_, the current urban vegetation cover decreases concentrations as may be expected due to the additional dry deposition, but the additional conversion of open urban greenspace to woodland is forecast to increase concentrations in urban areas, although slight reductions are seen away from sources (figure 3). This would imply that tree planting, aimed at reducing NO_2_ emissions, may in fact increase urban NO_2_ concentrations through processes that are explored in §3c(iii).

**Table 5.** Summary of the effect of current vegetation on concentrations averaged over entire UK and grid cells dominated by urban landcover (calculated as current vegetation versus no urban vegetation) for 2015, together with the additional effect of 25 or 50% conversion of urban greenspace to urban woodland (calculated as additional urban tree cover versus current vegetation). Absolute concentrations are in µg m^−3^.

		reduction by current urban vegetation (UrbanBASE–NoUrbanVEG)		additional reduction by 25% urban tree planting (25OGSC–UrbanBASE)		additional reduction by 50% urban tree planting (50OGSC–UrbanBASE)	
		UK	urban	UK	urban	UK	urban
PM_10_	current vegetation	13.43	16.42	13.43	16.42	13.43	16.42
	change in concentration	−0.036	−0.11	−0.013	−0.049	−0.025	−0.10
	difference (%)	−0.27%	−0.69%	−0.093%	−0.30%	−0.18%	−0.59%
PM_2.5_	current vegetation	6.05	8.78	6.05	8.78	6.05	8.78
	change in concentration	−0.025	−0.084	−0.0098	−0.036	−0.019	−0.070
	difference (%)	−0.42%	−0.95%	−0.16%	−0.41%	−0.32%	−0.80%
BSOA	current vegetation	0.19	0.20	0.19	0.20	0.19	0.20
	change in concentration	+0.00077	+0.0018	+0.00025	+0.00045	+0.00050	+0.00091
	difference (%)	+0.41%	+0.90%	+0.13%	+0.23%	+0.26%	+0.45%
SO_2_	current vegetation	0.74	1.61	0.74	1.61	0.74	1.61
	change in concentration	−0.013	−0.098	−0.0016	−0.0083	−0.0032	−0.016
	difference (%)	−1.70%	−5.77%	−0.22%	−0.52%	−0.44%	−1.02%
NH_3_	current vegetation	1.46	1.89	1.46	1.89	1.46	1.89
	change in concentration	−0.012	−0.080	−0.00069	−0.0023	−0.0014	−0.0044
	difference (%)	−0.80%	−4.07%	−0.047%	−0.12%	−0.092%	−0.23%
NO_2_	current vegetation	4.39	13.87	4.39	13.87	4.39	13.87
	change in concentration	−0.026	−0.20	+0.0011	+0.021	+0.0022	+0.043
	difference (%)	−0.61%	−1.41%	+0.025%	+0.15%	+0.051%	+0.31%
O_3_	current vegetation	71.52	64.30	71.52	64.30	71.52	64.30
	change in concentration	−0.16	−0.94	+0.025	+0.12	+0.051	+0.24
	difference (%)	−0.23%	−1.44%	+0.035%	+0.18%	+0.071%	+0.37%

### (c) Assessment of interactions

#### (i) Wet deposition

The modelling of the pollutant capture by vegetation through the ACTM approach allows some interesting interactions to be identified and quantified that may not be entirely obvious initially. The reduction in pollutant concentration due to the enhanced dry deposition to vegetation results in a reduction in wet deposition, which to some extent counteracts the benefit (table 6). The relative effect is largest for compounds that are primarily deposited via wet deposition such as PM. In the national vegetation scenarios, for example, an increase in PM_2.5_ dry deposition of 19.9 kt yr^−1^ is accompanied by a decrease in wet deposition by 10.4 kt yr^−1^. Thus, the net removal is actually only half of what would be estimated by considering dry deposition in isolation, e.g. through the i-Tree approach. For PM_10_, the net removal is only two-thirds of what would be expected. The interaction with wet deposition is to some extent accounted for in the calculation of the Emission Damage Costs that underpin the deposition-based evaluation. This is derived by modelling the increase in concentration and exposure for changes in concentrations and wet deposition is presumably considered in this evaluation. However, precipitation varies between years both in magnitude and spatial distribution, and the importance of the interaction, therefore, highlights an important uncertainty in the deposition-based approach.

**Table 6.** Summary of the dry and wet deposition under the national UKBASE and NoVEG (no vegetation) scenarios, together with the net change in deposition and the fractional importance of the wet deposition correction, for 2015. All absolute deposition amounts are in kt yr^−1^.

	dry deposition			wet deposition			
	NoVEG	UKBASE	Δ dry	NoVEG	UKBASE	Δ wet	Δ net dep
PM_10_	236.4	275.6	+39.2	1317.3	1304.3	−13.0	+26.1 (−33%)
PM_2.5_	40.7	60.6	+19.9	241.4	231.0	−10.4	+9.5 (−52%)
SO_2_	10.7	29.3	+18.6	12.0	11.3	−0.7	+17.9 (−4%)
NH_3_	19.9	55.2	+35.3	29.4	25.9	−3.5	+31.8 (−10%)

#### (ii) Emissions

An increase in vegetation cover is associated with an increase in emissions of biogenic volatile organic compounds (BVOC) which themselves are a precursor for ozone and PM_2.5_ formation. This is a recognized negative side effect of tree planting [20–22]. However, vegetated soils also tend to emit more NO than bare soil and this contributes to the net effect of vegetation on NO_2_ concentrations being negligible. In a world devoid of vegetation, soil resuspension would be increased. This effect adds to the benefits of vegetation for PM_2.5_ and PM_10_.

#### (iii) Chemistry

Chemical interactions occur at various places in the modelled atmospheric system. Changes in the chemical pollution climate affect the dry deposition rates of pollutants. This tends to be only crudely represented by current deposition modelling approaches. The dry deposition of SO_2_ and NH_3_ in the EMEP model depends on the annual average NH_3_/SO_2_ ratio [23,24], i.e. in highly alkaline environments, SO_2_ deposition is enhanced and NH_3_ deposition is limited and *vice versa* [13]. Thus, chemical interactions not only occur in the atmosphere, but also on leaf surfaces.

Another route of chemical interactions is through air chemistry. For example, because vegetation enhances the terrestrial sink for all pollutants (except BVOCs and soil NO), many secondary pollutants tend to be decreased not only through their increased removal, but also due to the increased removal of their chemical precursors, exacerbating the vegetation's overall effect.

The response of NO_2_ to vegetation changes seen in the simulations is less intuitive and requires further consideration: UK vegetation decreases NO_2_ in urban areas, but increases it in more remote regions. For the urban runs, current urban vegetation again decreases NO_2_ in urban areas, but additional conversion to urban forest is simulated to increase NO_2_. As mentioned before, at the UK scale, conversion to forest in the EMEP4UK model was configured to increase the soil NO emission in the model, and, although this increased total NO*x* emission by only a small amount, this is a possible reason why NO_2_ increases in the rural environment by vegetation. However, in the urban runs, this increase in soil NO emission was deactivated and still additional tree planting increased NO_2_. In addition to sources and deposition sinks, NO_2_ concentrations depend on the photo-stationary equilibrium between NO, O_3_ and NO_2._

For the urban scenarios, current total urban vegetation has the effect of reducing O_3_, while the conversion of open urban greenspace to urban woodland is simulated to increase O_3_ everywhere in the UK (electronic supplementary material, figure S1; table 5). The reason is found in the relative magnitudes of the increased terrestrial sink compared with the increase in O_3_ production due to larger BVOC emissions from trees. Total urban vegetation includes trees that emit BVOCs, but the increase in the dry deposition sink has the larger impact on O_3_ concentrations. Additional conversion of open urban greenspace to urban forest increases O_3_ production (through increased BVOC emissions) more than it reduces O_3_ through more efficient dry deposition. The increased O_3_ concentration in the tree planting scenarios results in increased titration of NO as can be seen in reduced NO concentrations across the country (electronic supplementary material, figure S2) and this is partly responsible for the increase in the NO_2_ concentration. However, the maps of total NO*x* (=NO + NO_2_) (electronic supplementary material, figure S3) show almost exactly the same pattern as NO_2_, suggesting that the re-partitioning between NO and NO_2_ via O_3_ interaction cannot be the sole or even main reason for the increase in NO_2_. An additional two model scenario runs were performed to shed light on the underlying processes: these were a current vegetation base run and a 50% open urban greenspace conversion run in both of which BVOC emissions were switched off. The results show that without the change in BVOC emissions associated with tree planting, the increase in urban tree cover causes a very small (less than 0.3%) reduction in NO*x* concentrations in urban areas, which is due to the only small difference in deposition rates between grassland and forest in the model (electronic supplementary material, table S4). At the same time, the NO/NO_2_ partitioning changes slightly in response to changes in O_3_ deposition and concentration. Thus, it is the additional BVOC emission in the urban tree planting scenarios which causes the increase in NO_2_ in the model through two additive effects: firstly, the BVOC emissions increase NO*x* concentrations (presumably by competing for OH and reducing NO*x* oxidation to HNO_3_), and secondly, they also increase O_3_, which in turn modifies the NO/NO_2_ partitioning in favour of NO_2_.

## 4. Discussion

### (a) Magnitude of air pollution removal

The scope for pollution removal by vegetation is of particular interest for PM, which tends to dominate the health impacts of air pollution, and for O_3_, which causes health impacts in particular in polluted, warmer regions. While the current health burden of NO_2_ is estimated to be lower, this compound is currently in the spotlight because many cities across Europe are out of compliance with target values. Several studies have attempted to quantify the removal of air pollution by vegetation and, in particular, urban trees and vegetation, including in the UK, and estimates differ widely. Here, we show that this is in large part a question of scale. The (UK) national vegetation as a whole reduces air pollution levels significantly, estimated here at 10% for PM_2.5_, 30% for SO_2_, 24% for NH_3_ and 13% for O_3_. Importantly, this includes the cumulative effect of pollutant removal during transport from source to receptor. For example, the concentration in London is lowered also by the uptake of some of the pollutants transported long range from continental Europe by vegetation encountered *en route*, e.g. in Kent. It also includes the secondary effect of reducing precursor gases involved in secondary PM and O_3_ formation.

By contrast, the effect of localized vegetation on local concentrations is limited: the overall effect of urban vegetation on urban PM_2.5_ is a reduction of the order of 1% and a conversion of 50% of available open urban greenspace to urban forest would add a further reduction of similar magnitude. By comparison, applying a simpler model in which total PM had to be scaled up from the primary fraction to two example UK conurbations, McDonald *et al*. [25] estimated that a 50% planting scenario might lower PM_10_ deposition by 4% in the English Midlands and 0.7% in Glasgow, which compares to an average urban reduction of 0.6% derived here. In the earlier study, the urban cells were defined via the city boundary and in the Midlands scenario included agricultural areas situated between the cities of Birmingham and Coventry, with an increased potential for planting, and this explains the higher gain. Litschke & Kuttler [26] similarly estimated a typical PM_10_ reduction by 1% due to trees in urban areas, while Nowak [27] estimated that current vegetation removed less than 1% of PM_10_ and NO_2_ in Chicago and would only remove less than 5% if the city was fully covered by trees. The new study is also consistent with conclusions of the UK's Air Quality Expert Group that, while beneficial, urban tree planting is not a solution for reducing urban air pollution at the city scale [28].

Many alternative studies of valuing air pollutant removal by vegetation have been based on quantifying the benefit via the quantification of dry deposition rather than through modelling the effect on the concentration itself, including, for example, for the vegetation in London [29,30], but few studies appear to have taken the ACTM approach adopted here.

The assessment of the human health benefit is not the primary topic of this paper, but these can be estimated using the results of an assessment based on the total urban vegetation by Jones *et al*. [8]. According to that study, existing UK urban woodland removes 0.7 kt PM_2.5_ yr^−1^, reducing the health burden from PM_2.5_ by about 1900 life years lost/year, with a similar gain achievable through the 50% planting action. The PM_2.5_ removal of a single mature tree would then equate to 1.7 life hours/year saved.

### (b) Implications for micro-intervention and green infrastructure

Although the pollutant collection properties of small green infrastructure interventions depend on the exact set-up and location, the country-level model results enable some approximate downscaling to investigate the efficacy that may be expected.

#### (i) Approximate PM_2.5_ collection efficiency of a single tree

Commercial forests are typically planted at a density of 1000–2000 trees ha^−1^, but a fully mature woodland has a final density of closer to 100 trees ha^−1^ [31]. Tree surveys for London and other cities suggest that most urban trees have a diameter at breast height (DBH) of less than 20 cm [30]. At this DBH, one might expect a higher planting density, more like 400 trees ha^−1^, for a closed forest stand.

The current UK urban woodland extent was estimated here to be 976 km^2^ and to take up 0.70 kt PM_2.5_ yr^−1^ (electronic supplementary material, table S5) [8]. Assuming full maturity, this equates to a removal of 71 g PM_2.5_ mature tree^−1^ yr^−1^, about 64 g yr^−1^ more than if the same area were covered by grassland.

To put this into context, the UK National Atmospheric Emissions Inventory implies a total (i.e. exhaust plus non-exhaust) fleet average combined (all road types) emission factor for diesel cars of 0.023 g PM_2.5_ km^−1^ [32]. At a typical annual travel distance of about 15 000 km yr^−1^ [33], a car emits 350 g PM_2.5_ yr^−1^, probably somewhat more under urban driving conditions, similar to the uptake of five properly mature trees. A taxi on average covers almost three times this mileage (14 mature tree equivalents). For buses, the total fleet average emission is 0.094 g PM_2.5_ km^−1^. In London, 490 million km are covered annually by local buses with a total fleet of about 10 000 vehicles [34]. Thus, based on these average figures, each London bus travels on average about 50 000 km yr^−1^, thus generating an emission of 4.7 kg PM_2.5_ yr^−1^, the offsetting of which would require 73 mature trees, or about 3/4 ha of mature urban woodland.

The emission from domestic solid fuel burning has recently come under increased scrutiny. In the UK, larger biomass boilers have a strict emission limit of 30 g PM/GJ in order to be eligible for government subsidy under the UK's Renewable Heat Incentive (RHI) [35]. At a domestic heat requirement of 5000–30 000 kWh (18–108 GJ), such appliances might emit up to 0.54–3.2 kg PM yr^−1^, mainly as PM_2.5_. Older appliances and those not covered by the RHI may emit significantly more [36]. Room heaters are generally less efficient: even the EC Ecodesign Directive (2009/125/EC) allows a maximum emission of 5 g PM kg^−1^ dry matter burnt for log stoves [37]. Assuming a room heater burns 1 tonne of dry wood a year, it would cause an emission of up to 5 kg PM yr^−1^, mainly in the form of PM_2.5_, similar to the average bus above, but emitted over a much smaller fraction of the year (and concentrated on individual days of the week and hours of the day). This emission is again equivalent to the PM removal of some 78 trees or 0.78 ha of woodland. Older appliances and open fireplaces not adhering to the Ecodesign standard are likely to emit significantly more, and so does the use of non-ideal fuels (e.g. wood with high moisture content) [38].

#### (ii) Artificial trees

A number of solutions have been developed as green infrastructure solutions to reducing air pollution. These range from passive uptake such as green walls or green lampposts to active filtering devices such as the commercial CityTree [39,40]. According to the advertising material on the company website, the CityTree may be run with an airflow rate of 5.5 m^3^ min^−1^ and removes 15 ± 5, 23 or 26–64% of PM_2.5_, depending on the study. The absolute amount removed from the air scales with air concentration. In the UK, the modelled annual average urban concentration of PM_2.5_ was 8.8 µg m^−3^. Even at a significantly more polluted UK location with an average PM_2.5_ concentration of 25 µg m^−3^, the device would remove just over 20 g PM_2.5_ yr^−1^, based on an average efficiency value of 28%, about one-quarter of what is estimated here for a single mature tree. Of course, in a highly polluted location, e.g. in some Asian cities, the concentrations are much larger and the CityTree would remove much more material, but then so would a tree.

#### (iii) Large-scale green infrastructure projects

Because it would be lacking the mechanical aspiration of the CityTree, a passively collecting green infrastructure element of similar size, such as a green wall or a green lamppost, would likely remove significantly fewer pollutants. Turbulent transport limitations would hamper the pollutant uptake. Green walls and roof gardens have been suggested as features for large-scale introduction of green infrastructure into urban areas. In China, first cities are planned as green cities from inception. The 50% urban woodland simulation has shown that conversion of 12% of the total urban area (table 1) from open urban greenspace into urban woodland would reduce urban emissions of PM_2.5_ by about 0.8% on average (table 5). Given that roads, car parks and other sealed areas that cannot be converted to green infrastructure make up a significant fraction of the urban space, even a large-scale implementation of green infrastructure would be unlikely to make a much larger impact than the 50% tree planting scenario and this assumes that the PM_2.5_ capture efficiency of the green infrastructure matches that of mature trees.

It should be noted, however, that the pollutant removal by green infrastructure depends on the exact positioning of the infrastructure and that ACTM modelling cannot simulate the intricacies between individual surface elements and ground-level concentration. Using an atmospheric chemistry model expanded with an extension to simulate street canyon mixing and dry deposition, Pugh *et al*. [41] suggested that continuous green wall cover within street canyons in particular may be effective in reducing street-level concentrations of PM by up to 60% while highlighting the need to get the design right to avoid concentration increases. This is a very extreme value for 100% vegetation cover, assuming deep canyons, very low wind speeds (1 m s^−1^ at roof level) and relatively large deposition velocities that are likely to be inconsistent with these low wind speeds. Furthermore, this green infrastructure would ameliorate mainly the concentration build-up deriving from in-canyon sources rather than the contribution from background concentrations and will, therefore, mainly aid the reduction in roadside increments. Nevertheless, that study highlights that certain infrastructure options may offer opportunities to reduce local source contributions. Consistent with our study, Pugh *et al*. [41] found that green rooftops were relatively ineffective in reducing street-level concentrations. Overall, our findings mirror those of Vos *et al*. [42] who argue that localized urban vegetation cannot be expected to be a solution for alleviating local air pollution hot-spots.

### (c) Implications for deposition-based approaches of valuing air pollution removal by vegetation

This study reveals important interactions and feedbacks that can be quantified by the full ACTM approach, but which are not taken into account when the pollutant removal benefit is quantified via quantifying the deposition via approaches based on prescribed concentrations that do not account for deposition-concentration feedbacks. The negative side effect of BVOC emissions through tree planting on air pollution has been widely recognized through their role in increasing O_3_ concentrations [20]. The present study highlights an additional, secondary effect on NO_2_ levels and shows that if tree species are not carefully selected, urban tree planting could even increase concentrations of NO_2_ and, therefore, one of the key pollutants they are often meant to reduce. This implies a greater than 100% error in static deposition-based approaches which would predict a benefit while the real net effect may actually be negative.

Clearly, the exact magnitude of the chemical effect on urban NO_2_ depends on the additional BVOC emission associated with the landcover change. In the EMEP model, the additional urban tree cover is ascribed the same isoprene and monoterpene emission factors as is thought to be representative for the average existing UK vegetation. If trees were selected to minimize BVOC emissions (e.g. [20,21,43]), it may be possible to control the effect on O_3_ and NO_2_.

Furthermore, the results highlight the magnitude of the counteracting effects on wet deposition. As increased dry deposition removal lowers air concentration, this in turn reduces wet deposition. This offsetting effect is here found to be large (50%) for slowly depositing compounds such as PM_2.5_, and introduces considerable uncertainty when quantifying the benefit via the deposition term and emission damage costs. Clearly, the relative magnitude depends on the amount of precipitation in the area and is likely to be larger in the UK than in some other parts of the world. This is an issue that does not appear to have so far been fully recognized in deposition-based approaches like i-Tree Eco. For local-scale interventions, the enhancement in dry deposition and reduction in wet deposition become spatially decoupled, adding to the uncertainty in assigning geographically varying damage costs to deposition increases.

### (d) Wider impacts of urban vegetation

#### (i) Co-benefits of vegetation

Overall, the scope of reducing pollution levels through urban vegetation is shown to be very limited. Nevertheless, it may be worthwhile considering and valuing this effect within the context of the multiple other (ecosystem) services provided by vegetation in general and urban vegetation in particular. The benefit of vegetation for air pollution removal, even if modest, might provide one of several elements for the decision to go ahead with green infrastructure measures.

The overall benefits and disbenefits of tree planting have been summarized in several review papers [22,44,45]. For example, the i-Tree Eco model also ascribes a value to the role of urban trees for carbon sequestration, their assistance in water management by reducing storm-water run-off and their effect on heating/cooling [12,30]. This latter effect can be beneficial or detrimental: trees generally reduce the heat-island effect, which in summer potentially increases labour productivity and reduces adverse health outcomes (morbidity and mortality) and/or reduces air conditioning needs; but trees may increase heating bills in winter [46]. Similarly, at the local level, trees can shelter houses from wind or they can shade buildings with contrasting effects on energy requirements in winter. Green spaces reduce noise, promote physical activity and contribute significantly to the wellbeing of the society and provide long-term benefits to physical and mental health [47,48], which have also been speculated to improve the immune response to air pollution [48,49] and may, therefore, be beneficial in combating air pollution effects through a second route.

Semi-natural ecosystems and especially also urban vegetation such as gardens support a large number of plant and animal species. However, the benefits of vegetation for biodiversity are more difficult to value economically as there is little consensus on the value society ascribes to biodiversity *per se*, beyond its role in ecosystem provisioning [50].

#### (ii) Disbenefits of vegetation

Where urban vegetation is targeted at reducing air pollution, negative side effects also need to be considered as well as the positives:

*Other pollutant emissions.* Some pollutant emissions associated with vegetation (e.g. pollen) and its management are not considered in this study: wounding of leaves and branches due to grass cutting or pruning emits leaf alcohols and other VOCs with potentially large ozone and BSOA forming potential, even for plant species that are generally low VOC emitters [51]. Fertilizer application may result in increased emissions of ammonia (NH_3_) which is involved in secondary aerosol formation and has detrimental effects on biodiversity and plant species adapted to low-nitrogen growing conditions, nitric oxide (NO) as well as nitrous oxide (N_2_O), a potent greenhouse gas [52]. Potential application of fungicides and pesticides has its own environmental impacts and an increase in the emissions of allergens like pollen need to be considered when evaluating the overall health impact of vegetation for the population [53].

*Safety.* There are costs involved in the management of vegetation to reduce dangers to the public. Road safety can be a significant concern when it comes to green infrastructure: if poorly managed, leaf fall can render roads, pavements and paths slippery and increase the risk of road accidents and falls. Falling trees and branches can cause injury to passersby, property and residents, and are one of the main cause of storm-related deaths [54]. In addition, UK city councils have expressed their concerns about the potential effect the increase of tree cover in urban parks might have on crime and personal safety, and leaf fall from deciduous trees can clog up drainage of urban run-off.

Thoughtful species selection and careful management can reduce many of these disbenefits.

In addition to taking up air pollutants, trees can affect local turbulence and wind speed, which can also have adverse effects on concentrations. Above forests, the increased aerodynamic roughness of trees compared with short vegetation and asphalt/concrete tends to increase vertical mixing and dispersion of pollutants and this tends to reduce surface concentrations. The situation is very different within the tree canopy and within the built-up environment: street canyon trees can significantly lower the air flow and dispersion of local emissions, depending on local conditions and meteorology [55]. As a result, urban trees can increase ground-level concentrations and human exposure to air pollutants at the same time as promoting their removal through enhanced dry deposition. For example, using CFD simulations, Jeanjean *et al*. [56] found for a 4 km^2^ city area in Leicester, UK, that dispersion had a negative effect that exceeded the enhanced deposition by an order of magnitude at low wind speed, while at higher wind speeds, it had a positive effect that again exceeded the positive effect of dry deposition. Trees can, at the same time, increase the accumulation of pollutants on one side of the street canyon, while lowering concentration on the other [22,45]. Trees are sometimes planted to protect individual receptor sites, e.g. at school carparks. Here, again, the effect can be positive or negative, depending on geometry and meteorology [42]. The literature is more consistent about the use of trees for separating people from sources, e.g. through hedge-like features between roads and pavements [21,22]. In this situation, the trees increase the line of travel of pollutants from source to receptor and promote dispersion and dilution *en route*. While such interventions might increase concentrations at the source side, they are highly likely to reduce concentration at the receptor side, independent of meteorology [22]. The use of vegetation to spatially separate people from sources appears to be their least controversial and most effective use. However, it should be mentioned that non-vegetation features such as walls would physically have the same effect.

### (e) Uncertainties

Uncertainties in the modelling work are significant and difficult to quantify. Both the ACTM- and deposition-based approaches are sensitive to the parametrization of the dry deposition process used. These are uncertain and vary significantly. For example, Flechard *et al*. [57] compared the results from four deposition models, including the EMEP parametrization, to derive dry deposition of reactive nitrogen compounds from time-integrated concentration measurements across 55 European sites. This exercise indicated an intra-model variability in the deposition velocities (*V*_d_) of a factor of 3 for NH_3_ and 5 for NO_2_. For fine aerosol components (NH_4_^+^, NO_3_^−^), relative good consistency (within a factor of 2) was found for grass and semi-natural short vegetation (shrubland), but values varied by more than a factor of 10 for forest. This variability reflected not only the parametrization of the deposition mechanism, but also the model assumptions on tree height and leaf area index. The EMEP model tends to fall into the middle of the range for most compounds, but it is one of two models that predicts significantly lower aerosol *V*_d_ to forest than the other two models, whose values look unreasonably large in the light of more recent reviews of aerosol dry deposition [58].

Additional uncertainty arises from the limitations of the chemistry scheme in the model. In particular, many aspects of the mechanisms leading to BSOA formation are uncertain: as is typical for ACSMs the EMEP emissions model and chemical scheme only treats some key BVOCs. In particular, very rapid BSOA formation from sesquiterpenes and some monoterpenes may be missing [59]. In addition, as mentioned before, the simulations here assume the same average tree species mix as currently implemented in the EMEP model, while in the urban scenarios, in particular the BVOC emissions will depend on the tree species that exist or are planted in the urban context.

## 5. Conclusion

Quantification of the benefits of vegetation appears to be an emotive subject and estimates of the efficacy of vegetation and in particular trees to combat air pollution vary greatly in the literature. This study shows that this is at least in part a question of scale of the vegetation extent. While total national vegetation together significantly reduces pollution levels (e.g. about 10% for PM_2.5_), urban vegetation is estimated to account for only a small reduction in pollution levels and even very large-scale conversion of available open urban greenspace to urban forest would reduce urban air concentrations by only about 1% overall. The impact of small-scale green infrastructure implementations on air quality is very small, except where the vegetation acts as a barrier between source and receptor, and in most cases far less economic than implementing measures to reduce emissions in the first place. Thus, the benefit of urban tree planting for air pollution should in general only be considered as one of multiple benefits within the natural capital approach.

The use of an ACTM to estimate reductions in concentrations, exposure and associated health impacts, while more computationally cumbersome, has key advantages over the more commonly used deposition approach in which air pollution removal by vegetation is valued via the damage cost of the equivalent emission (e.g. i-Tree Eco): it is mass conserved and accounts for physical and chemical interactions. The UK-wide simulations identified that a purely dry-deposition-based approach would overestimate the PM_2.5_ uptake by UK vegetation by a factor of 2, if the associated reduction in wet deposition is ignored. Increased natural BVOC and soil NO emission from forest ecosystems further counteract the benefit of the vegetation for PM_2.5_ (via BSOA formation), NO*_x_* and also O_3_. The simulations suggest that vegetation only has a small effect on NO_2_ (and NO*x*) at the national scale and that conversion of urban green space to urban forest may even increase NO_2_ levels. This could be traced to the combined impact of increased BVOC emissions on increasing NOx levels and, by also increasing O_3_, sustaining more of the NO*x* as NO_2_. Thus, the magnitude of the increase depends on the BVOC emission potential of the tree species selected, but even for non-emitters, the potential of NO_2_ removal through urban forests was found to be negligible.

## Supplementary Material

Supplementary Material

## Data accessibility

The modelled annual average concentration and deposition fields from which the statistics presented here are derived are available from the NERC Environmental Informatics Data Centre (EIDC) at https://eidc.ac.uk/ at https://catalogue.ceh.ac.uk/documents/bad6721c-574b-4229-b023-c7b13ae4c099. The data on pollution removal by UK vegetation are also available via an ONS data selector at https://www.ons.gov.uk/economy/environmentalaccounts/articles/ukairpollutionremovalhowmuchpollutiondoesvegetationremoveinyourarea/2018-07-30.
