# Supplementary material for: Potential and limitation of air pollution mitigation by vegetation and uncertainties of deposition-based evaluations
Source: Philos Trans A Math Phys Eng Sci. 2020 Sep 28;378(2183):20190320. doi: 10.1098/rsta.2019.0320 (PMC7536036; doi:10.1098/rsta.2019.0320)
Supplement: Supplementary Material [file rsta20190320supp1.docx]

**Potential and limitation of air pollution mitigation by vegetation and uncertainties of deposition-based evaluations**


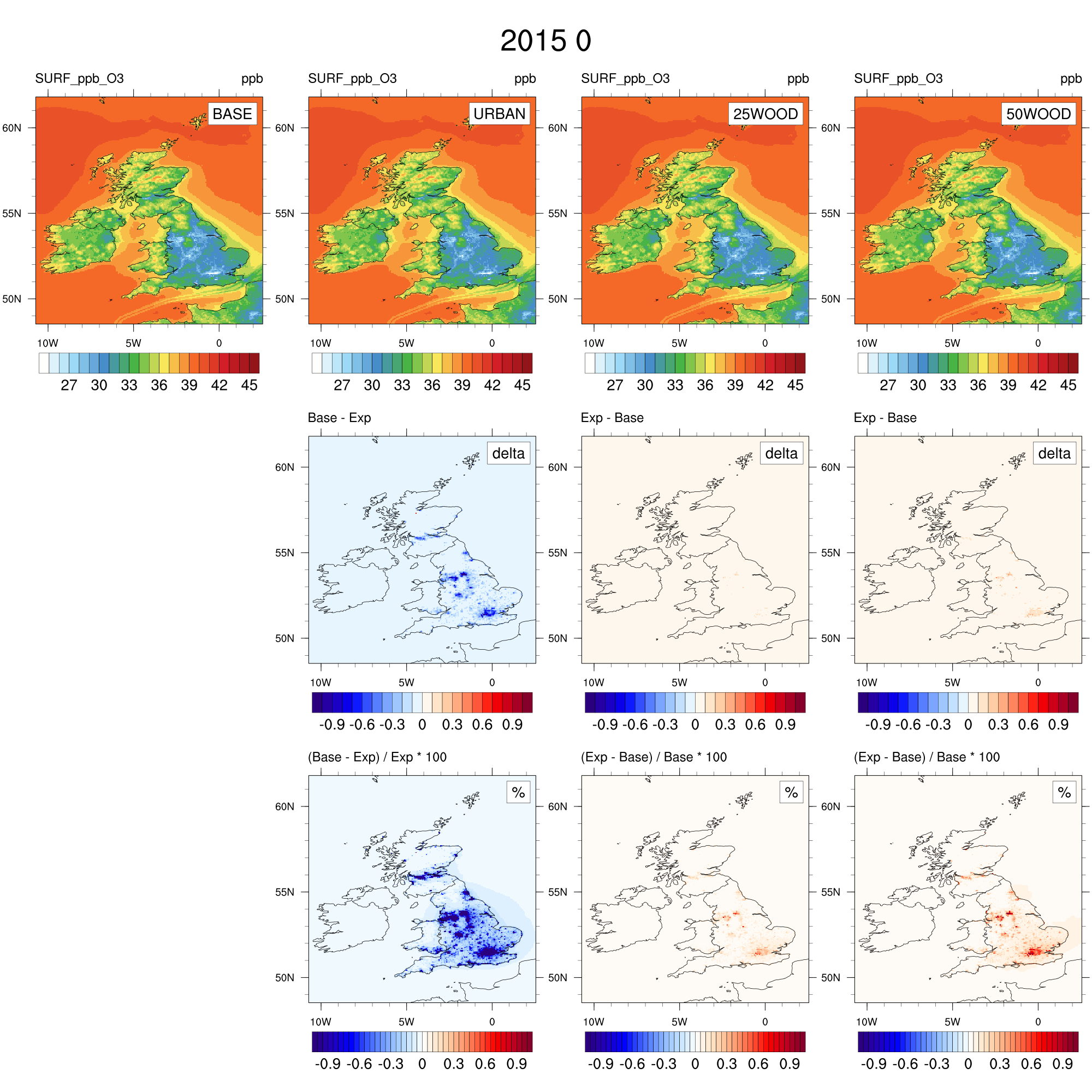


(c)

(a)

(b)

**Supplementary Figure 1:** Maps for O_3_ for 2015, showing (a) the annual average NO_2_ concentration to a vegetation-less UK [µg m^-3^], together with its (b) absolute and (c) relative change due to UK vegetation (UKBASE-NoVEG), with red (blue) values indicating an increase (decrease) in concentration above vegetation compared with no-vegetation.


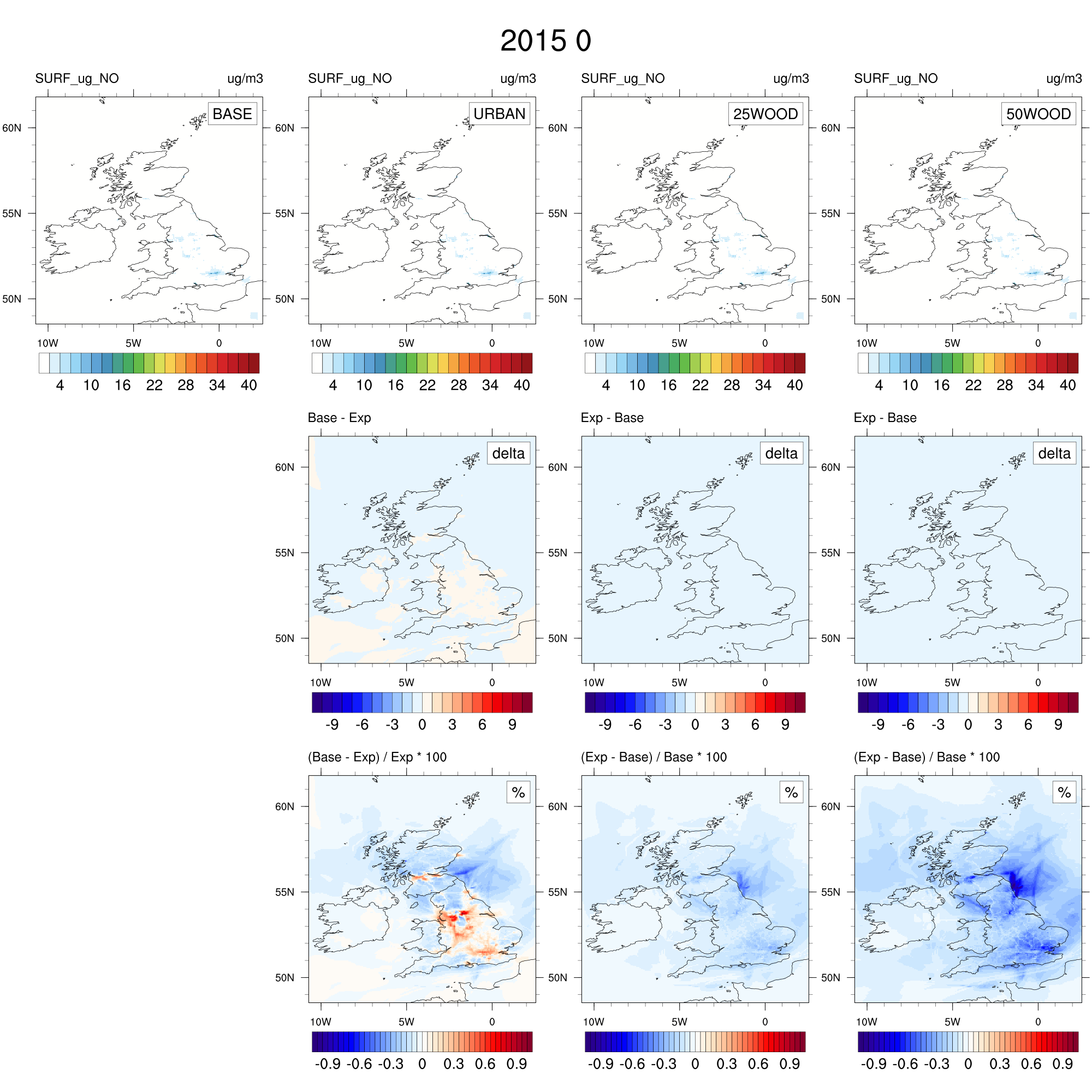


(c)

(b)

(a)

**Supplementary Figure 2:** Maps for NO for 2015, showing (a) the annual average NO_2_ concentration to a vegetation-less UK [µg m^-3^], together with its (b) absolute and (c) relative change due to UK vegetation (UKBASE-NoVEG), with red (blue) values indicating an increase (decrease) in concentration above vegetation compared with no-vegetation.


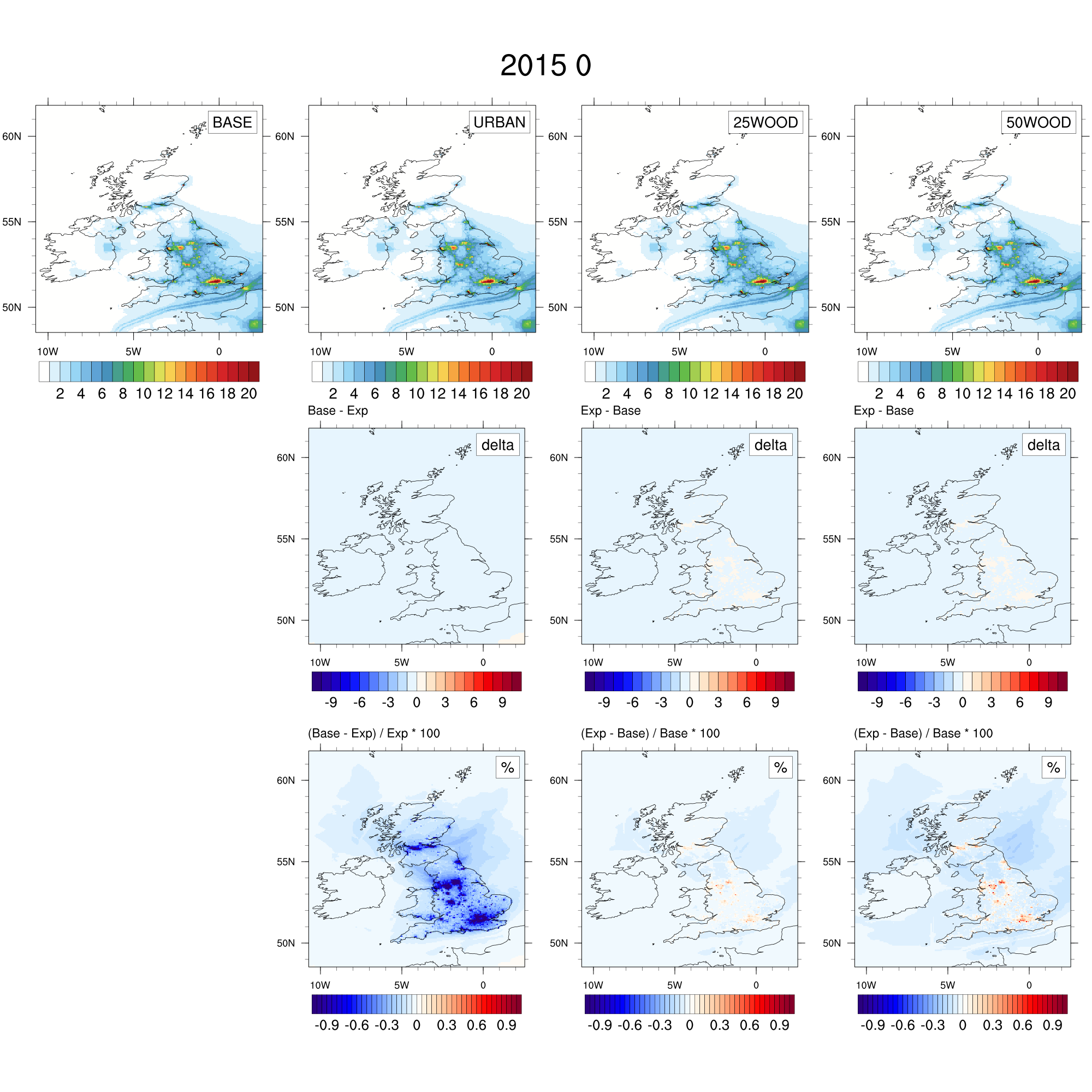


(c)

(b)

(a)

**Supplementary Figure 3:** Maps for NO_x_ for 2015, showing (a) the annual average NO_2_ concentration to a vegetation-less UK [µg m^-3^], together with its (b) absolute and (c) relative change due to UK vegetation (UKBASE-NoVEG), with red (blue) values indicating an increase (decrease) in concentration above vegetation compared with no-vegetation.

**Supplementary Table 1.** Annual average concentrations of PM under current and no-vegetation landcover scenarios and effect of change in concentration relative to national no vegetation scenario for different years. wb dust: windblown dust from desert soil. BSOA: biogenic secondary organic aerosol. The urban average is also for the national no-vegetation runs, but calculated over all grid cells for which the urban landcover types jointly account for at least 50%.

| **Pollu-tant** | **Scenario** | **2007** | | **2011** | | **2015^*^** | | |
| --- | --- | --- | --- | --- | --- | --- | --- | --- |
|  |  | **UK** | **urban** | **UK** | **urban** | **UK** | | **urban** |
| **Non-dust PM_10_** | Current vegetation | 11.55 | 14.40 | 10.74 | 13.20 | 9.90 | 11.84 | |
|  | No vegetation | 12.54 | 15.61 | 11.60 | 14.19 | 10.55 | 12.60 | |
|  | Change in concentration | -0.98 | -1.21 | -0.86 | -0.99 | -0.65 | -0.77 | |
|  | **Difference (%)** | **-7.82%** | **-7.73%** | **-7.41%** | **-7.00%** | **-6.15%** | **-6.07%** | |
| **Non-dust PM_2.5_** | Current vegetation | 6.36 | 8.93 | 6.08 | 8.25 | 4.85 | 6.79 | |
|  | No vegetation | 7.20 | 10.01 | 6.83 | 9.14 | 5.40 | 7.46 | |
|  | Change in concentration | -0.84 | -1.08 | -0.75 | -0.89 | -0.55 | -0.67 | |
|  | **Difference (%)** | **-11.7%** | **-10.7%** | **-11.0%** | **-9.73%** | **-10.2%** | **-8.94%** | |
| **wb dust PM_10_** | Current vegetation | 0.063 | 0.066 | 0.033 | 0.021 | 0.11 | 0.10 | |
|  | No vegetation | 1.19 | 1.28 | 0.22 | 0.13 | 2.00 | 1.87 | |
|  | Change in concentration | -1.13 | -1.22 | -0.19 | -0.11 | -1.89 | -1.77 | |
|  | **Difference (%)** | **-94.7%** | **-94.9%** | **-83.9%** | **-84.0%** | **-94.6%** | **-94.4%** | |
| **wb dust PM_2.5_** | Current vegetation | 0.016 | 0.016 | 0.0095 | 0.0069 | 0.026 | 0.025 | |
|  | No vegetation | 0.28 | 0.29 | 0.055 | 0.036 | 0.46 | 0.43 | |
|  | Change in concentration | -0.26 | -0.28 | -0.046 | -0.029 | -0.44 | -0.40 | |
|  | **Difference (%)** | **-94.3%** | **-94.4%** | **-82.9%** | **-80.9%** | **-94.1%** | **-94.2%** | |
| **PM_2.5_ BSOA** | Current vegetation | 0.22 | 0.25 | 0.21 | 0.25 | 0.16 | 0.18 | |
|  | No vegetation | 0.17 | 0.19 | 0.15 | 0.18 | 0.10 | 0.12 | |
|  | Change in concentration | +0.050 | +0.055 | +0.060 | +0.063 | +0.056 | +0.056 | |
|  | **Difference (%)** | **+29.4%** | **+28.5%** | **+40.0%** | **+34.1%** | **+60.0%** | **+44.8%** | |

* Model run performed with 2014 emissions and 2015 meteorology.

**Supplementary Table 2.** Average annual concentrations of a range of gaseous pollutants under current and no-vegetation landcover scenarios and effect of change in concentration relative to no vegetation scenario. Absolute concentrations are in µg m^-3^.

| **Pollu-tant** |  | **2007** | | **2011** | | **2015^*^** | |
| --- | --- | --- | --- | --- | --- | --- | --- |
|  | **Scenario** | **UK** | **urban** | **UK** | **urban** | **UK** | **urban** |
| **SO_2_** | Current vegetation | 1.46 | 4.01 | 1.07 | 2.76 | 0.85 | 2.00 |
|  | No vegetation | 2.07 | 4.90 | 1.55 | 3.43 | 1.21 | 2.49 |
|  | Change in concentration | -0.61 | -0.89 | -0.48 | -0.67 | -0.36 | -0.49 |
|  | **Difference (%)** | **-29.5%** | **-18.2%** | **-31.0%** | **-19.5%** | **-29.8%** | **-19.8%** |
| **NH_3_** | Current vegetation | 1.32 | 2.04 | 1.49 | 2.28 | 1.33 | 2.02 |
|  | No vegetation | 1.78 | 2.59 | 1.95 | 2.79 | 1.74 | 2.48 |
|  | Change in concentration | -0.46 | -0.55 | -0.51 | -0.51 | -0.41 | -0.46 |
|  | **Difference (%)** | **-25.8%** | **-21.2%** | **-23.6%** | **-18.4%** | **-23.6%** | **-18.5%** |
| **NO_2_** | Current vegetation | 9.33 | 26.74 | 7.62 | 21.01 | 5.80 | 17.06 |
|  | No vegetation | 9.55 | 27.76 | 7.69 | 21.54 | 5.80 | 17.41 |
|  | Change in concentration | -0.22 | -1.02 | -0.070 | -0.53 | 0.000 | -0.30 |
|  | **Difference (%)** | **-2.30%** | **-3.67%** | **-0.91%** | **-2.48%** | **0.00%** | **-2.00%** |
| **O_3_** | Current vegetation | 67.67 | 57.18 | 66.85 | 59.08 | 70.58 | 64.04 |
|  | No vegetation | 80.20 | 66.28 | 79.43 | 68.37 | 82.83 | 73.33 |
|  | Change in concentration | -12.53 | -9.10 | -12.58 | -9.29 | -12.24 | -9.29 |
|  | **Difference (%)** | **-15.6%** | **-13.7%** | **-15.8%** | **-13.6%** | **-14.8%** | **-12.7%** |

* Model run performed with 2014 emissions and 2015 meteorology.

**Supplementary Table 3:** Comparison of different metrics aimed at estimating the change in concentration in urban areas for PM_2.5_ and PM_10_.

| **Pollutant** | **Averaging method** | **current veg**  **UrbanBASE** | **no urban veg**  **(NoUrbanVEG)** | | **25% tree  planting  (25OGSC)** | | **50% tree planting**  **(50OGSC)** | |
| --- | --- | --- | --- | --- | --- | --- | --- | --- |
| PM_10_ | UK | 13.43 | -0.036 | -0.27% | -0.013 | -0.09% | -0.025 | -0.18% |
|  | UK, population weighted | 16.32 | -0.10 | -0.61% | -0.042 | -0.25% | -0.083 | -0.51% |
|  | urban areas | 16.42 | -0.11 | -0.69% | -0.049 | -0.30% | -0.10 | -0.59% |
|  | urban, population weighted | 16.77 | -0.12 | -0.68% | -0.048 | -0.29% | -0.10 | -0.57% |
|  | rural, population weighted | 14.94 | -0.056 | -0.37% | -0.021 | -0.14% | -0.041 | -0.28% |
| PM_2.5_ | UK | 6.05 | -0.025 | -0.42% | -0.0097 | -0.16% | -0.019 | -0.32% |
|  | UK, population weighted | 8.64 | -0.073 | -0.84% | -0.031 | -0.35% | -0.060 | -0.70% |
|  | urban areas | 8.78 | -0.084 | -0.95% | -0.036 | -0.41% | -0.070 | -0.80% |
|  | urban, population weighted | 9.04 | -0.085 | -0.93% | -0.035 | -0.39% | -0.070 | -0.77% |
|  | rural, population weighted | 7.40 | -0.040 | -0.54% | -0.016 | -0.21% | -0.031 | -0.42% |

**Supplementary Table 4.** Summary of effective mean deposition velocities (*V*_d_) (mm s^-1^) extracted from model outputs as a spatial and temporal average over the year, for each pollutant, by landcover type. These were calculate as *V*_d_ = - average annual flux to a given landcover type / average concentration and then averaged over all grid cells.

| Pollutant | Habitat | 2007 | 2011 | 2015 |
| --- | --- | --- | --- | --- |
| PM_10_ | Coniferous woodland | 7.74 | 7.60 | 7.88 |
|  | Deciduous woodland | 5.23 | 5.15 | 5.31 |
|  | Crops | 2.15 | 2.10 | 2.35 |
|  | Moorland/ grassland | 2.34 | 2.30 | 2.54 |
|  | Water | 2.12 | 2.05 | 2.37 |
|  | Desert | 2.15 | 2.08 | 2.34 |
| PM_2.5_ | Coniferous woodland | 6.35 | 6.22 | 5.94 |
|  | Deciduous woodland | 4.02 | 3.82 | 3.71 |
|  | Crops | 0.71 | 0.59 | 0.66 |
|  | Moorland/ grassland | 0.96 | 0.94 | 0.91 |
|  | Water | 0.60 | 0.54 | 0.58 |
|  | Desert | 0.60 | 0.59 | 0.57 |
| SO_2_ | Coniferous woodland | 16.06 | 18.24 | 17.77 |
|  | Deciduous woodland | 15.89 | 18.1 | 17.68 |
|  | Crops | 5.05 | 5.51 | 5.45 |
|  | Moorland/ grassland | 6.92 | 7.65 | 7.55 |
|  | Water | 6.41 | 6.47 | 6.75 |
|  | Desert | 1.15 | 1.26 | 1.17 |
| NH_3_ | Coniferous woodland | 15.00 | 12.99 | 12.81 |
|  | Deciduous woodland | 14.63 | 12.63 | 12.52 |
|  | Crops | 3.53 | 3.34 | 3.33 |
|  | Moorland/ grassland | 6.79 | 6.20 | 6.32 |
|  | Water | 6.5 | 6.27 | 6.6 |
|  | Desert | 1.2 | 1.27 | 1.25 |
| NO_2_ | Coniferous woodland | 0.97 | 0.93 | 1.00 |
|  | Deciduous woodland | 0.72 | 0.67 | 0.77 |
|  | Crops | 0.54 | 0.51 | 0.54 |
|  | Moorland/ grassland | 0.53 | 0.52 | 0.53 |
|  | Water | 0.05 | 0.05 | 0.05 |
|  | Desert | 0.05 | 0.05 | 0.05 |
| O_3_ | Coniferous woodland | 4.44 | 4.34 | 4.32 |
|  | Deciduous woodland | 3.91 | 3.82 | 3.83 |
|  | Crops | 3.5 | 3.39 | 3.51 |
|  | Moorland/ grassland | 2.78 | 2.74 | 2.67 |
|  | Water | 0.49 | 0.49 | 0.5 |
|  | Desert | 0.5 | 0.5 | 0.5 |

**Supplementary Table 5:** Pollutant capture by urban green and blue space in the urban cross-cutting account, as dry deposition of pollutants (ktonnes per year) (from Jones et al., 2019).

| Pollutant | Habitat | kt/yr removed in Britain 2015 |
| --- | --- | --- |
| PM_10_ | Urban woodland | 1.23 |
|  | Urban open greenspace | 0.45 |
| PM_2.5_ | Urban woodland | 0.70 |
|  | Urban open greenspace | 0.31 |
| SO_2_ | Urban woodland | 0.59 |
|  | Urban open greenspace | 1.00 |
| NH_3_ | Urban woodland | 0.44 |
|  | Urban open greenspace | 0.95 |
| NO_2_ | Urban woodland | 0.41 |
|  | Urban open greenspace | 1.61 |
| O_3_ | Urban woodland | 4.97 |
|  | Urban open greenspace | 16.94 |
